# Estimation of the Difference in Colistin Plasma Levels in Critically Ill Patients with Favorable or Unfavorable Clinical Outcomes

**DOI:** 10.3390/pharmaceutics13101630

**Published:** 2021-10-06

**Authors:** Jose Sanabria, Vivian Garzón, Tatiana Pacheco, Maria-Paula Avila, Julio-Cesar Garcia, Diego Jaimes, Angela Torres, Rosa-Helena Bustos, Javier Escobar-Perez, Deisy Abril

**Affiliations:** 1 Evidence-Based Therapeutic Group, Clinical Pharmacology, Universidad de la Sabana, Chía 140013, Colombia; josesanva@unisabana.edu.co (J.S.); viviangaru@unisabana.edu.co (V.G.); tatiana.pacheco@unisabana.edu.co (T.P.); mariaaviga@unisabana.edu.co (M.-P.A.); julio.garcia@unisabana.edu.co (J.-C.G.); diegojf@unisabana.edu.co (D.J.); torresangelag@gmail.com (A.T.); 2 Doctoral Programme of Biosciences, Universidad de La Sabana, Chía 140013, Colombia; 3 Laboratorio de Genética Molecular Bacteriana, Universidad El Bosque, Bogotá 110121, Colombia; djabril@unbosque.edu.co

**Keywords:** therapeutic drug monitoring (TDM), colistin, multi-drug resistance bacteria, Gram-negative bacteria

## Abstract

In recent decades, antimicrobial resistance (AMR) has led to an increased use of therapeutic alternatives. Among these options, colistin continues to be an option for the treatment of multi-resistant (MDR) Gram-negative bacterial infections. However, due to its high toxicity (nephrotoxicity and neurotoxicity) and narrow therapeutic window, colistin treatment must be utilized carefully. Colistin-treated patients have been observed to have higher mortality due to inadequate therapeutic levels. The objective of this study was to estimate the difference in colistin plasma levels in critically ill patients, and its relationship to favorable or unfavorable clinical outcomes. This prospective observational study was conducted between September 2017 and June 2020 at the Universidad de La Sabana Clinic, in patients who had been treated with colistimethate sodium (CMS) for at least 72 h until day 7 of drug treatment in the critical care unit of a university hospital. There were no statistically significant differences in colistin levels between groups with favorable or unfavorable clinical outcomes (0.16 SD vs. 0.54 SD *p*-value = 0.167). There was higher mortality in patients with subtherapeutic levels (18% vs. 0%), and additionally, there was a greater rate of renal failure in the group with higher therapeutic levels (50% vs. 20.7%). Due to the loss of power of the study, we were unable to demonstrate a possible difference between colistin levels related to favorable or unfavorable clinical outcomes at day 7. However, we recommend further studies to evaluate the impact of measuring levels in terms of mortality and security.

## 1. Introduction

Antimicrobial resistance (AMR) has been reported in bacteria for thousands of years, yet the magnitude of the problem is only now being recognized [1]. Dozens of studies have now shown that an increase in AMR brings with it a substantial increase in morbidity, mortality, and reduced quality of life [2,3,4]. Each year, 50,000 deaths occur secondary to AMR. If the indiscriminate use of antibiotics does not improve, the World Health Organization has estimated that by 2050, approximately 10 million people will die as a result of AMR [5]. The economic burden will be considerable, particularly due to additional hospitalizations, expensive secondary treatment options, and productivity losses in the labor market [6]. In 2014, the Center for Disease Control and Prevention (CDC) estimated that 50% of antibiotics are unnecessarily prescribed in the US, with an estimated cost of 1.1 trillion dollars [7]. Despite the serious consequences of the inappropriate use of antibiotics and alarming future projections, the use of antibiotics has increased by 4–5% across different contexts [6].

The AMR problem includes all families of bacteria. According to the World Health Organization (WHO), more than 50% of bacteria, including *Escherichia coli*, *Klebsiella pneumoniae* and *Staphylococcus aureus*, have been reported as resistant to antibiotics around the world [8]. In 2008, a first list of pathogens with emerging risk of AMR was published, called the ESKAPE group, including *Enterococcus faecium*, *Staphylococcus aureus*, *Klebsiella pneumoniae*, *Acinetobacter baumannii*, *Pseudomonas aeruginosa*, and Enterobacter species [9]. The situation is even more worrying in Latin America. In 2009, the SENTRY antimicrobial surveillance program in Latin America showed higher AMR levels compared to other regions [10]. Some efforts have been made in Colombia to address the AMR burden, but there are still gaps to be addressed. One report from public organizations in the country revealed that the alarming indiscriminate use of antibiotics is leading to a high incidence of multi-drug resistant (MDR) bacterial infections, at a cost of around USD 191,000 [11,12,13].

International consensus has defined an even greater problem within the Gram-negative bacteria group, due to restricted therapeutic options for these bacteria. The latest-generation of cephalosporins cannot be used as empirical therapies in many countries; therefore, carbapenems remain the first-line therapy around the world, generating accelerated resistance, particularly in intensive care units (ICUs), where the risks of selective pressure and transmission are higher [9]. No new classes of antimicrobials have been discovered or patented since 1987 [5], and the lack of investment by the pharmaceutical industry in new antibiotic development due to reduced profit margins and regulatory obstacles hinders effective solutions [14]. This situation has led to a resurgence of ancient antibiotics, such as the polymyxin family, for the treatment of infections caused by MDR.

Colistin has been clinically available since the 1950s, but it soon fell into disuse, mainly due to its nephrotoxic potential [15]. Alongside its nephrotoxicity, which ranges between 0 and 58% in different studies [16,17], some evidence has found the incidence of peripheral neuropathy may be up to 50% with its use, as the main manifestation of neurotoxicity [18]. The lack of other available options has increased the use of colistin in MDR infections, even with its safety profile [19]. The pharmacokinetics of colistin are complex. Colistin is available as a sulfate and as sodium colistimethate (CMS). Colistin sulfate is formulated only as a topical product, and is not orally absorbable. CMS is a prodrug that is administered intravenously or inhaled, and is rapidly hydrolyzed to produce several metabolites, including the active ingredient colistin. Colistin binds strongly to lipid membranes of the liver, kidney, lung, brain, heart, and muscle cells by electrostatic interactions [20]. Pharmacokinetic data on CMS and colistin are scarce. CMS has a half-life of 124 min, while colistin as the active ingredient has a half-life of 251 min [21]. Colistin has a calculated volume of distribution of 0.34 L/kg. CMS is excreted in urine, and colistin is excreted by non-renal mechanisms. Biliary excretion in humans has not been documented to date [21]. The distribution of colistin to the pleural cavity, lung parenchyma, and cerebrospinal fluid is relatively low [22], and it has even been documented that the concentrations reached in the central nervous system are not bactericidal. Therefore, it can be administered by inhalation or intrathecally to achieve optimal concentrations [23].

Despite the number of clinical reports of the successful use of colistin against MDR infections caused by *P. aeruginosa*, *A. baumannii*, or *K. pneumoniae*, there is a dearth of information on its pharmacokinetics, pharmacodynamics, and toxicodynamics; such information is essential to establish optimal dosing regimens [24,25]. One of the strategies for optimizing dosing regimens is therapeutic drug monitoring (TDM). The benefits of TDM are best demonstrated for antibiotics with a narrow therapeutic index, particularly aminoglycosides and vancomycin. The traditional TDM approach for these antibiotics was primarily aimed at avoiding toxicity. However, recent developments in TDM for other antibiotics, such as Beta-lactams, quinolones, linezolid, or daptomycin, with their broader therapeutic indices, suggest a valid use for TDM in outcomes related to efficacy [26]. Regarding colistin, TDM includes microbiological bioassays [27,28], Fourier-transform infrared spectroscopy (FTIR) [29,30], high-resolution liquid chromatography (HPLC) [31,32], liquid chromatography–mass spectrometry (LC-MS) [33,34,35], and ultra-performance liquid chromatography–electrospray tandem mass spectrometry with electrospray ionization (UPLC-ESI-MS/MS) [36]. The HPLC technique has been widely used due to its robustness, sensitivity, and accuracy compared to other techniques, such as bioassays. To date, there remains a gap in the identification of the optimal plasma concentrations of colistin to guarantee efficacy and low toxicity. Recent studies have shown that the current CMS dose regimens are associated with suboptimal colistin concentrations, which are far from meeting pharmacokinetic targets for many strains of multidrug-resistant Gram-negative bacilli [37]. In addition, no clinical studies have evaluated the possible difference of colistin plasma concentrations among patients with and without favorable clinical outcomes [38].

Taking into account that the literature regarding the therapeutic monitoring of colistin is scarce, the absence of a globally validated method for its measurement, the great variability of the clinical results regarding efficacy and safety, and the few prospective studies published, we conducted a prospective study aiming to determine whether there are differences in colistin plasma concentrations between patients with and without favorable clinical outcomes.

## 2. Materials and Methods

### 2.1. Study Design, Setting and Subjects

This prospective observational study was conducted from September 2017 and June 2020 at a university clinic in the Hospitalization and Critical Care services. The participation of patients in the study was according to the proposed inclusion criteria. Patients over 18 years old, hospitalized for infections caused by multiresistant Gram-negative carbapenemase-producing bacteria, diagnosed in hospital at the clinic of the University of La Sabana, who had been treated with CMS for at least 7 days at the institution, were included. Patients were excluded if they were pregnant or lactating women, empirical use of CMS was not indicated, they had a previous diagnosis of acute and/or chronic renal failure, their samples were in inadequate condition for the proper measurement of colistin levels, or if control of the infectious focus with drainage or surgical intervention had not been carried out during the first 72 h of treatment. Detailed demographic (age, sex, weight), clinical (site of infection, severity of disease, comorbidities (Charlson comorbidity index), CMS loading dose, CMS maintenance dose, duration of treatment, days of stay in the hospital, mortality, carbapenemase class, concomitant antibiotic therapy), laboratory (hemoglobin, red blood cell, neutrophil and platelet counts, serum creatinine and glomerular filtration rate (GFR)), and microbiological (bacterial isolation and characterization) data from each patient were recorded.

The doses were prescribed by the treating physician based on the institution’s drug-therapeutic guidelines (load of 1 mg/kg and maintenance of 3 mg/kg per day, divided into three doses). Measurement of the colistin concentration in plasma was performed on the fourth day of treatment, when it was assumed that the colistin concentrations would have reached a steady state. Colistin plasma concentrations (C_min_) were measured just before CMS administration. C_min_ was chosen because it is more convenient from a practical point of view. Clinical outcomes were classified as (a) favorable (absence of fever (T < 38 °C); leukocytosis <12,000; not requiring vasopressors; in infections in which control cultures could be obtained, these cultures must be sterile within 7 days of treatment and without changes in the initial antimicrobial regimen within of the first 7 days of treatment. In addition, mortality at 30 days was measured as a secondary outcome; or (b) unfavorable (in the case of fever > 38 °C; leukocytosis > 12,000; requirement for a vasopressor or the presence of positive control cultures for the initial microorganism). The clinical outcomes were determined by consensus of at least two physicians on the study team. If all the criteria were met, the patient was assigned as having a favorable outcome; if one criterion was not met, the outcome was considered not favorable.

### 2.2. Determination of the Plasma Colistin Concentrations

Venous blood samples (approx. 3 mL) were collected using an EDTA tube (Vacutainer BD ^®^; Beckton Dickinson & Company, Franklin Lakes, NJ, USA) after four days of colistin treatment. The samples were centrifuged and chilled, and the plasma was stored at −20 °C until assayed. Concentrations of colistin in the plasma of patients were determined using a modified HPLC method [39,40,41,42,43], Supplementary Materials File S1. The Ethics Committee of the Universidad de La Sabana Clinic approved this study (Act 12 September 2017), and signed written informed consent was obtained from all patients who participated in the study. Universidad de La Sabana, grant number MED-222-2017 and Departamento Administrativo de Ciencia, Tecnologıa e Innovacion, MinCiencias (grant number 123080763958.

### 2.3. Bacterial Isolates and Detection of Resistant Genes

The characterization of the isolates was carried out from pure cultures, which were previously isolated from each sample in the clinical laboratory. This laboratory performs all the cultures following the guidelines of the manual for taking samples for microbiological analysis of the Bogotá District Secretariat, which is a consensus that takes into account the international references. Therefore, in addition to the Gram staining, multiple agars such as blood or chocolate were used depending on the type of sample, in aerobic and anaerobic environments to search for multiple pathogenic species.

The bacterial isolates were recovered in MacConkey agar (Oxoid-Thermo Scientific^®^, Hampshire, UK) and sent to the Bacterial Molecular Genetics Laboratory in AMIES transport media (COPAN). MacConkey agar medium was used to grow the isolates prior to DNA extraction, and the MIC broth microdilution assay isolates were cryopreserved at −80 °C in TSB (Oxoid-Thermo Scientific^®^, Hampshire, UK), supplemented with glycerol 15%. Bacterial identification and susceptibility profiles to meropenem, doripenem, imipenem, ceftazidime, cefoxitin, cefepime, trimethoprim/sulfamethoxazole, piperacillin/tazobactam, gentamicin, amikacin, ciprofloxacin, and tigecycline were determined by automated VITEK^®^2 systems, using the breakpoints defined by the Clinical and Laboratory Standards Institute [44]. The MICs of meropenem, amikacin, and colistin were established by the broth dilution method on a microplate. *E. coli* ATCC^®^ 25922^TM^ and *P. aeruginosa* ATCC^®^ 27853^TM^ strains was used as a susceptibility control (American Type Culture Collection). Finally, the carbapenemase genes *bla*_IMP_, bla_OXA-48_, bla_VIM_, bla_GES_, bla_KPC_, and bla_NDM_ were assessed by multiplex PCR [45]. 

### 2.4. Statistical Analysis

The sample size calculation was performed through EPIDAT 3.1 (Odense M, Denmark, Europe), by the module for testing hypotheses in studies that compare two means of two independent groups. Normality test was perform using Shapiro–Wilk test. The median with IQR was used for continuous variables, categorical data were expressed as numbers and percentages, and a comparison of variables was made between the groups of unfavorable clinical outcome and favorable clinical outcome, and for continuous variables using the Wilcoxon test and for categorical variables using the Chi-square test. A *p*-value < 0.05 was required to achieve statistical significance. All analyses were performed using STATA. 14.0.5 (StataCorp. 2015. Stata Statistical Software: Release 14. College Station, TX: StataCorp LP, USA).

## 3. Results

**Figure 1:**
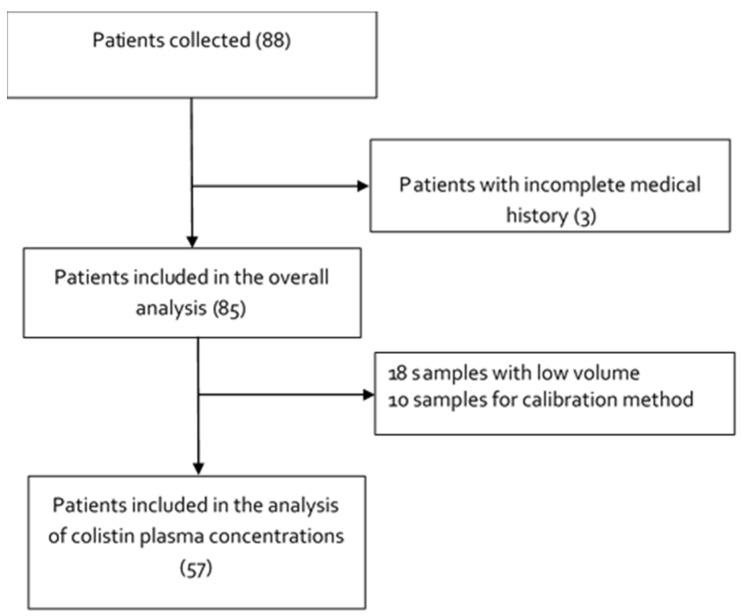
Study flow diagram.

From April 2007 to April 2020, 88 samples from patients treated with colistin were taken. Of the 88 patients, 3 had to be excluded for reasons highlighted in Figure 1. Ten samples were used for method optimization. Colistin levels were measured in 57 samples; 18 samples could not be evaluated as the sample volume was not enough to be injected in triplicate onto the HPLC (Figure 1).

### 3.1. Demographic Characteristics

Table 1 summarizes the demographic characteristics of the 85 patients included in this study. Of the patients included, 64 (72.7%) were male. The median ± standard deviation age was 59 (interquartile range, IQR: 45–75), and the body mass index (BMI) was 25.5 (IQR: 21.3–29). The median number of days of hospitalization was 30 days (IQR 23–43). 

An exploratory bivariate analysis was performed in order to determine if there were differences between groups according to clinical outcome, with statistical significance for sex, SOFA score day 1, SOFA score day 7, APACHE II score, CCI, and mortality. Multivariate analysis based on logistic regression was calculated; it did not show association between the variables and the outcome, except for SOFA score day 7 (Supplementary Materials File S3). The selection of patients was not considered in the design when performing bivariate analysis, as can be seen in the asymmetry of the groups obtained (Supplementary Materials File S3). 

### 3.2. Clinical Outcome

Table 1 shows the clinical outcomes (favorable and unfavorable). The outcome was favorable in 58.8% of patients. The median age for the favorable outcome group was 59 years, body mass index (BMI) was 25.2, leukocytes on day 1 were 10,860, and leukocytes on day 7 of follow-up were 8970. The median number of days of hospitalization was 29. The other 41.2% of patients had an unfavorable outcome. The median age for the unfavorable outcome group was 60 years, body mass index (BMI) was 25.6, leukocytes on day 1 were 13,000, and leukocytes on day 7 of follow-up were 1715. The median number of days of hospitalization was 32. There was a mortality rate of 4% at 30-day follow-up for patients with a favorable outcome, and of 34.28% in the group with an unfavorable outcome. 

### 3.3. Clinical Prognosis Scales

For the total population, the median SOFA score at day 1 was 4, and at day 7 was 2. The median APACHE scale at baseline was 10, and the Charlson Comorbidity Index (CCI) was 2. For the group with a favorable clinical outcome the median SOFA score was 3.5 at day 1, and 2 at day 7. The median APACHE scale was 8, and the Charlson Comorbidity Index was 2. For the group with an unfavorable outcome, the median SOFA score at day 1 was 4 and at day 7 was 4.5. The median APACHE scale was 12, and the median Charlson Comorbidity Index was 4 (Table 1). 

### 3.4. Site of Infection and Microbiological Isolation

The most common resistance pattern for the different microorganisms isolated was serine-type carbapenemases (KPC) in 76.4%, followed by the double detection of KPC and metallo-beta-lactamases (VIM) in 11.76%. Detection of only VIM occurred in 7.05% of the patients.

The most common site of infection was the urinary tract with 28.2% of cases, followed by the respiratory tract (23.5%), abdominal infection (20%), bacteremia (15.2%), bone and joints (8.2%), and both central nervous system and skin (2.35%). In the group with favorable clinical outcomes, urinary tract infections were more common (30% vs. 25.7%), while for patients with unfavorable clinical outcomes, respiratory tract infections were more common (31.4% vs. 18%). For the remaining sites of infection, there was no difference.

The most frequently isolated bacteria in the total study population were: *Pseudomonas aeruginosa* (61.1%), followed by *Klebsiella pneumoniae* (14.1%), *Enterobacter cloacae* (10.5%), *Pseudomonas putida* (4.7%), *Klebsiella oxytoca* and *Escherichia coli* (3.52%), and both *Acinetobacter baumannii* and *Providencia rettgeri* (1.1%). In the group with favorable clinical outcomes, it was more common to find *Pseudomonas aeruginosa*, while in the group with unfavorable clinical outcomes it was more common to find *Klebsiella pneumoniae* (Table 1).

Table 2 shows the complete susceptibility profile from the molecular genetics laboratory of 41 isolates from the study. *P. aeruginosa* isolates were the only isolates with resistance to β-lactams, polymyxins, aminoglycosides, quinolones, and glycyclines. Of the 30 isolates shown, 16 (53.3%) presented resistance to colistin, which had not been documented before by the clinic’s laboratory, of which 8 (26.7%) were resistant to all five families of antibiotics evaluated. Regarding the isolates that were recovered from patients with unfavorable outcomes, the percentages of resistance were higher in all cases. *Klebsiella pneumoniae* isolates were resistant to β-lactams, polymyxins, and aminoglycosides; of the five isolates, four (80%) were only resistant to β-lactams and one (33.3%) was resistant to colistin and aminoglycosides. The *P. putida* isolates recovered from favorable outcome patients were resistant to β-lactams and quinolones, while the isolates from unfavorable outcome patients were only resistant to β-lactams. The *A. baumannii* isolate was resistant to β-lactams, aminoglycoside, and quinolones, the *E. cloacae* isolate was resistant to β-lactams and colistin, *C. freundii* was resistant to β-lactams and quinolones, and *E. coli* only to β-lactams.

Table 3 shows the total prevalence of isolates and the percentage of carbapenemase detection for some of them. In *P. aeruginosa*, the presence of *bla*_KPC_ was observed in 10% of isolates, *bla*_VIM_ in 6.7%, and 56.7% presented both *bla*_KPC_ and *bla*_VIM_. Some of the *Klebsiella pneumoniae* isolates had *bla*_KPC_ (80%); 25% of *P. putida* harbored *bla*_VIM_ and 11.1% of *E. cloacae* and 33% of *E. coli* presented *bla*_KPC_. Taking into account the differences in both the number and expression of carbapenemases, an analysis of the minimum inhibitory concentration (MIC) to meropenem was performed. Interestingly, 56.7% of isolates of *P. aeruginosa* and one isolate of *P. putida* had a meropenem MIC >1024 µg/mL, of which 88.2% harbored the two genes of the carbapenemases KPC and VIM. This was contrary to the findings for *A. baumannii* (64 μg/mL), *K. pneumoniae*, and *E. cloacae*, whose highest MICs for meropenem were 32 μg/mL. Regarding the MIC to colistin, 53.3% of isolates of *P. aeruginosa* and one isolate of *K. pneumoniae* and *E. cloacae* were resistant, of which 94.4% had an MIC of 4 μg/mL. Finally, the MIC to amikacin was established in 23.3% of isolates of *P. aeruginosa*, one of *K. pneumoniae*, and one of *A. baumannii*, with 128 μg/mL being the highest MIC for *P. aeruginosa* and 64 μg/mL for the other species. This demonstrated that these antibiotics are still optimal therapeutic options for these resistant isolates.

### 3.5. Management with Colistin and Concomitant Antimicrobial Therapy

Only 57% of the patients received loading doses of colistin; the loading dose depended on medical criteria based on the patient’s age, weight, and renal function. Fifty percent of patients with a favorable outcome received loading doses, while 65.7% of patients with an unfavorable clinical outcome received loading doses. All patients received an initial maintenance dose of 1 mg/kg every 8 h.

Regarding concomitant antimicrobial therapy, the most prescribed antimicrobials together with colistin were: doripenem 63.5%, meropenem 27%, tigecycline 8.2%, and fosfomycin 1.1%. There was a higher prescription of doripenem in patients with a favorable clinical outcome (70% vs. 54.28%), and a higher prescription of meropenem in patients with an unfavorable clinical outcome (40% vs. 18%), as shown in Table 4.

### 3.6. Relationship between Colistin Levels and Favorable Clinical Outcomes and Mortality

**Figure 2:**
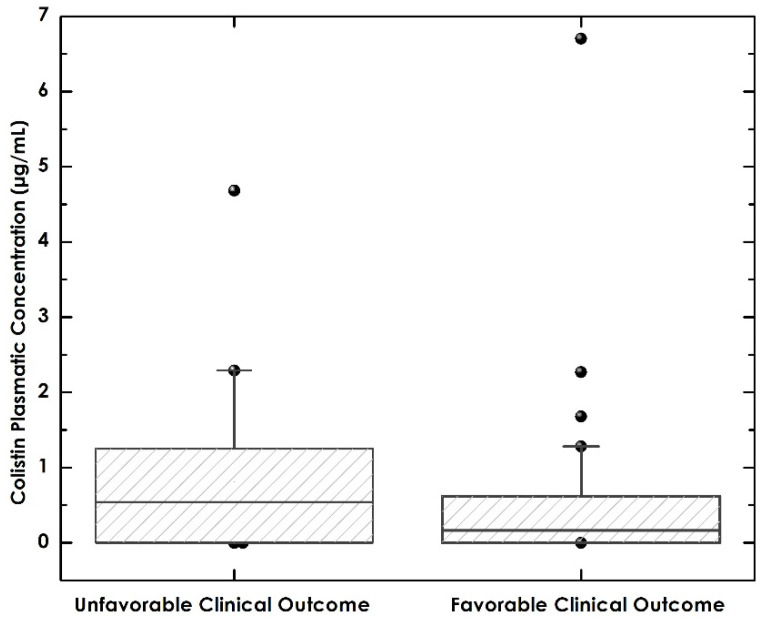
Levels of Colistin in plasma according to clinical outcomes.

The primary outcome was evaluated in 57 of the 88 patients, due to loss of samples during processing. The plasma colistin level for the general population was 0.51 (IQR 0–0.78). For the favorable outcome group, it was 0.16 (IQR 0–0.62), and for the unfavorable outcome group, it was 0.54 (IQR 0–1.25). There was no statistically significant difference between the two groups (*p* = 0.167) (Table 5 and Figure 2).

Of the 57 colistin samples tested, 53 were below the reference range (<2 µg/mL), 2 were in the normal range (2 to 4 µg/mL), and 2 were above the upper limit (>4 µg/mL). Favorable clinical outcomes occurred in 60% of patients with low colistin levels, 50% of patients with colistin levels within the normal range, and 50% of patients with levels above the limit of normality. Additionally, the percentage of mortality at 30 days was 18.8% for the group with low levels of colistin, and 0% for both normal levels and levels above the upper limit (Table 5).

Regarding the incidence of renal failure, defined as an increase in serum creatinine above 0.3 mg/dl in 48 h, this occurred in 20.7% of patients with low colistin levels, in 50% of patients with normal colistin levels, and in 50% of patients with high colistin levels (Table 5).

## 4. Discussion

In the present study, it was not possible to show that plasma colistin levels are related to a favorable or unfavorable clinical outcome in patients with infections of multiresistant Gram-negative bacteria. Colistin is a drug that has resurfaced as an alternative for the treatment of patients with multi-resistant bacterial infections, especially in countries with limited resources that do not have new antimicrobials for management; therefore, increasing the knowledge of the characteristics of this drug is indispensable to guaranteeing effective treatment. 

There are very few pharmacokinetic studies of colistin, since at the time of approval of this drug, the precise methodology for plasma measurement was not available. However, subsequent clinical studies have demonstrated changes in pharmacokinetic parameters in critically ill patients or patients on renal replacement therapy [46]. It has been recently reported that some patients may show variability in the pharmacokinetic profile of the drug, especially those patients who are in the intensive care unit or have acute or chronic renal failure [47]. This variability is related to therapeutic failure or increased risk of nephrotoxicity [48,49,50]. The therapeutic monitoring of drugs applied to colistin could be useful to maximize effectiveness and reduce toxicity; however, there is very little evidence in this regard. The concentrations tested in certain types of patients have been shown to be below the minimum recommended concentration [51], which can lead to treatment failure and increased mortality; additionally, it has been shown that trough levels are related to the incidence of nephrotoxicity in patients treated with colistin [52]. However, most of the articles available are case series or retrospective analyses, which are insufficient to fully clarify the relationship between plasma levels and the effectiveness and safety of colistin treatment. 

In this study, a significant difference in colistin plasma levels was not evidenced between patients with a favorable or unfavorable clinical outcome; however, mortality among patients with normal or high levels of colistin was 0%, compared to 18.8% among patients with low levels of colistin, indicating that the impact of colistin levels is more related to medium- or long-term outcomes than short term outcomes. These data must be interpreted with caution, however, since the population in the groups with normal or high levels of colistin may not have been representative of the general study population. Regarding safety, it was possible to identify that the group with high levels had a higher incidence of acute renal failure, although the population analyzed in this group consisted of only two patients. 

Routine clinical practice dose regimens, including those with higher doses, are in most cases not capable of achieving optimal colistin plasma concentrations; however, these levels are not associated with the steady-state equilibrium phase, clinical cure, or with crude mortality at day 30 [53]. Regarding the safety profile, TDM could be useful in the prevention of nephrotoxicity during colistin treatment [54]. Our study was not designed to demonstrate differences from a safety point of view; however, with these findings, we can hypothesize that there is a direct relationship between colistin levels and acute renal failure. Some authors have noted that the use of colistin without TDM might be unsafe, and guideline adherence does not warrant efficient target levels in critically ill patients [53].

Additionally, considering the baseline demographic characteristics of the patients, it was found that respiratory tract infections, isolation of multidrug-resistant Klebsiella pneumoniae, and the use of meropenem may be associated with a worse outcome in these types of patients. Although this study was not designed to find these differences, these findings are supported by other researchers. Rivera et al. found that the isolation of carbapenemase-producing *Klebsiella pneumoniae* is not considered a risk factor for mortality; however, they documented that this species may have high resistance to carbapenems in certain cases, which may explain the increased mortality in some patients [55]. Pardo et al. showed that the isolation of carbapenemase-producing *Klebsiella pneumoniae* and infection sites, especially respiratory tract or bloodstream infections, are related to higher mortality in patients with carbapenemase-producing Enterobacteriaceae infections [56]. Finally, Celis et al. found that pneumonia caused by carbapenemase-producing Enterobacteriaceae has higher mortality than other sites of infection by the same microorganisms [57]. The use of meropenem has not been described as a risk factor; the difference observed may be due to the fact that carbapenem, with the lowest MIC, has not been selected for combined therapy with colistin [58]. Furthermore, there was a statistically significant difference in the baseline characteristics of the patients: the SOFA and APACHE 2 prognostic scales were higher in the group with unfavorable clinical outcomes, showing a greater severity of disease. Understanding that colistin is a hydrophilic drug with low protein binding and mainly renal elimination suggests that this population could experience significant changes in the pharmacokinetics of the drug.

Regarding the limitations of the study, despite meeting N at the time of collection, when analyzing the clinical data of the 88 patients collected, we were only able to proceed with 85 patients, since 3 patients did not have complete data in the clinical database. Additionally, the analysis of plasma concentrations was carried out in only 57 patients, since 31 samples were lost in the method calibration process, which reduced the power of the study to find significant differences. We cannot know whether the patients not analyzed could be differentiated from the patients analyzed in the study. Furthermore, making conclusions based on population groups as small as two patients in the group with normal plasma levels, and two patients in the group with high plasma levels, is not possible.

Finally, despite the new guideline recommendations against the use of colistin, in countries with limited resources, it continues to be a therapeutic option of clinical relevance, making it necessary to determine whether there is a relationship between plasma levels and the safety and efficacy of multiresistant Gram-negative bacteria treatment. 

## 5. Conclusions

This study was unable to identify a significant difference between colistin levels and its relationship to favorable or unfavorable clinical outcome; larger studies are necessary to confirm the relationship between colistin levels and mortality over the medium to long term. Further research is also required to understand the incidence of renal failure according to plasma colistin levels, and the role of colistin TDM in the prognosis of patients.

## Figures and Tables

**Table 1:** Demographic characteristics and clinical outcomes for the 85 patients.

Variables	Included Patients (*n* = 85)	Favorable Outcome *n* = 50	Unfavorable Outcome *n* = 35	*p*-Value
Sex, (%), male	64 (72.72%) 21 (27.28%)	40 (80%)	21 (60%)	0.0438
Age (years), median (IQR)	59 (45–70)	59 (45–69.25)	60 (34–72)	0.8934
Site of infection (%)				
Urinary tract	24 (28.23%)	15 (30%)	9 (25.7%)	0.6657
Respiratory tract	20 (23.52%)	9 (18%)	11 (31.42%)	0.1509
Abdominal	17 (20%)	10 (20%)	7 (20%)	0.9999
Blood	13 (15.29%)	8 (16%)	5 (14.28%)	0.8289
Bone and joints	7 (8.23%)	4 (8%)	3 (8.57%)	0.9249
Skin	2 (2.35%)	2 (4%)	0 (0%)	NS
CNS	2 (2.35%)	2 (4%)	0 (0%)	0.2312
BMI, median (IQR)	25.56 (21.37–29.04)	25.27 (22.01–28.10)	25.63 (21.08–29.86)	0.5890
SOFA score day 1, median (IQR)	4 (2–6)	3.5 (2–4)	4 (3–8)	0.0131
SOFA score day 7, median (IQR)	2 (1–4.75)	2 (1–3)	4.5 (3–8)	0.0000
APACHE II, median (IQR)	10 (6.5–14)	8 (6–11)	12 (8–19)	0.0158
CCI, median (IQR)	2 (1–4)	2 (1–4)	4 (0–6)	0.0405
Days of hospitalization, median (IQR)	30 (23–43)	29 (21.75–39.25)	32 (25–45)	0.2698
GFR (mL/min) day 1, median (IQR)	93.5 (60–120.75)	95 (62–122)	89.5 (33–120)	0.4853
GFR (mL/min) day 7, median (IQR)	73 (35–103)	77 (37–116)	60 (28–103)	0.3309
Mortality (%)	14 (16.47%)	2 (4%)	12 (34.28%)	0.0002
Microorganisms found in isolation	*n*, (%)	Favorable *n*, (%)	Unfavorable *n*, (%)	
*Pseudomonas aeruginosa*	52 (61.17%)	34 (68%)	18 (51.42%)	
*Klebsiella pneumoniae*	12 (14.11%)	4 (8%)	8 (22.85%)	
*Acinetobacter baumannii*	1 (1.1%)	1 (2%)	0 (0%)	
*Klebsiella oxytoca*	3 (3.52%)	1 (2%)	2 (5.71%)	
*Escherichia coli*	3 (3.52%)	2 (4%)	1 (2.85%)	
*Enterobacter cloacae*	9 (10.58%)	6 (12%)	3 (8.57%)	
*Providencia rettgeri*	1 (1.1%)	0 (0%)	1 (2.85%)	
*Pseudomonas putida*	4 (4.7%)	2 (4%)	2 (5.71%)	
Colistin (%) Loading dose 1 mg/Kg	49 (57.64%)	25 (50%)	24 (65.71%)	

SOFA: Sequential Organ Failure Assessment; APACHE: Acute Physiology Additionally, Chronic Health Evaluation; CCI: Charlson Comorbidity Index; GFR: Glomerular Filtration Rate; CNS: Central Nervous System; NS: Not Specified, Supplementary Materials File S2.

**Table 2:** Susceptibility profile of some microorganisms included in the study.

Microorganism	Outcome	Antibiotic Family *n* (%)														
		β-Lactam			Polymyxins			Aminoglycoside			Quinolones			Glycylcyclines		
		S	I	R	S	I	R	S	I	R	S	I	R	S	I	R
*P. aeruginosa n* = 30	Favorable *n* = 10	0 (0)	0 (0)	10 (100)	2 (20)	4 (40)	4 (40)	2 (20)	0 (0)	8 (80)	2 (20)	0 (0)	8 (80)	0 (0)	0 (0)	9 (90)
	Unfavorable *n* = 14	0 (0)	0 (0)	14 (100)	1 (7.14)	6 (42.8)	7 (50)	1 (7.1)	0 (0)	13 (92.9)	2 (14.3)	0 (0)	11 (78.6)	0 (0)	0 (0)	11 (78.6)
*K. pneumoniae n* = 5	Favorable *n* = 3	0 (0)	0 (0)	3 (100)	0 (0)	1 (33.3)	2 (66.7)	2 (66.7)	0 (0)	1 (33.3)	2 (66.7)	0 (0)	0 (0)	1 (33.3)	0 (0)	0 (0)
	Unfavorable *n* = 2	0 (0)	0 (0)	2 (100)	2 (100)	0 (0)	0 (0)	2 (100)	0 (0)	0 (0)	1 (50)	1 (50)	0 (0)	2 (100)	0 (0)	0 (0)
*P. putida n* = 2	Favorable *n* = 1	0 (0)	0 (0)	1 (100)	0 (0)	1 (100)	0 (0)	0 (0)	1 (100)	0 (0)	0 (0)	0 (0)	1 (100)	0 (0)	1 (100)	0 (0)
	Unfavorable *n* = 1	0 (0)	0 (0)	1 (100)	1 (100)	0 (0)	0 (0)	1 (100)	0 (0)	0 (0)	1 (100)	0 (0)	0 (0)	1 (100)	0 (0)	0 (0)
*A. baumannii n* = 1	Favorable	0 (0)	0 (0)	1 (100)	0 (0)	1 (100)	0 (0)	0 (0)	0 (0)	1 (100)	0 (0)	0 (0)	1 (100)	1 (100)	0 (0)	0 (0)
*E. cloacae n* = 1	Favorable	0 (0)	0 (0)	1 (100)	0 (0)	0 (0)	1 (100)	1 (100)	0 (0)	0 (0)	1 (100)	0 (0)	0 (0)	1 (100)	0 (0)	0 (0)
*E. coli n* = 1	Favorable	0 (0)	0 (0)	1 (100)	0 (0)	1 (100)	0 (0)	0 (0)	1 (100)	0 (0)	1 (100)	0 (0)	0 (0)	1 (100)	0 (0)	0 (0)
*C. freundii n* = 1	Unfavorable	0 (0)	0 (0)	1 (100)	0 (0)	1 (100)	0 (0)	1 (100)	0 (0)	0 (0)	0 (0)	0 (0)	1 (100)	0 (0)	1 (100)	0 (0)

Abbreviations: S, susceptible; I, intermediate; R, resistant. Antibiotic tested: β-lactam (meropenem, doripenem, imipenem, ceftazidime, cefoxitin, cefepime, piperacillin/tazobactam), polymyxins (colistin), aminoglycoside (gentamicin, amikacin), quinolones (ciprofloxacin), and glycylcyclines (tigecycline). Antibiotics written in bold were further tested by broth microdilution.

**Table 3:** Prevalence of all microorganism and carbapenemase genes detected in the molecular genetics laboratory for some of them.

Microbiological Isolation	% of Prevalence	% *bla*_KPC_	% MBL		% *bla*_KPC_ + *bla*_VIM_	None
			*bla*_VIM_	Others		
*Acinetobacter baumannii*	1.13%	-	-	-		-
*Pseudomomonas aeruginosa*	59.1%	10%	6.7%	-	57%	-
*Escherichia coli*	3.4%	33%	-	-	-	-
*Enterobacter cloacae*	10.2%	11%	-	-	-	-
*Klebsiella oxytoca*	1.13%	-	-	-	-	-
*Klebsiella Oxytoca + E. coli*	1.13%	-	-	-	-	-
*Klebsiella pneumoniae*	13.6%	80%	-	-	-	-
*Klebsiella pneumoniae + Pseudomonas aeruginosa*	1.13%	-	-	-	-	-
*Providencia rettgeri*	1.13%	-	-	-	-	-
*Pseudomonas* sp.	1.13%	-	-	-	-	-
*Pseudomonas + Enterobacter cloacae*	1.13%	-	-	-	-	-
*Pseudomonas putida*	3.4%	-	25%	-	25%	-

**Table 4:** Concomitant medication.

Antibiotic	*n*, (%)	Favorable *n*, (%)	Unfavorable *n*, (%)
Doripenem	54 (63.5%)	35 (70%)	19 (54.28%)
Meropenem	23 (27%)	9 (18%)	14 (40%)
Tigecycline	7 (8.2%)	5 (10%)	2 (5.71%)
Fosfomycin	1 (1.1%)	1 (2%)	0 (0%)

**Table 5:** Primary and secondary clinical outcome.

Primary Outcome	General Population *n* = 57	Favorable *n* = 34	Unfavorable *n* = 23	*p*-Value
Colistin levels, Median, IQR	0.51 (0–0.78)	0.16 (0–0.62)	0.54 (0–1.25)	0.1670
Secondary outcome	Low colistin levels *n* = 53	Normal colistin levels *n* = 2	High colistin levels *n* = 2	
30 days mortality, *n*, %	10 (18.86%)	0 (0%)	0 (0%)	
Acute kidney injury, *n*, %	11 (20.75)	-	1 (50%)	

## Data Availability

The data presented in this study are available on request from the corresponding author. The data is not publicly available because it is stored in the research system of the Therapeutic Evidence group.

## Supplementary Materials

The following are available online at https://www.mdpi.com/article/10.3390/pharmaceutics13101630/s1, Supplementary Materials File S1. Analytical methodology for quantification of Colistin in Plasma, and Supplementary Materials File S2. Description of Clinical Scales, Supplementary Materials File S3. Statistical analysis
