# Supplementary material for: Estimation of the Difference in Colistin Plasma Levels in Critically Ill Patients with Favorable or Unfavorable Clinical Outcomes
Source: Pharmaceutics. 2021 Oct 6;13(10):1630. doi: 10.3390/pharmaceutics13101630 (PMC8540821; doi:10.3390/pharmaceutics13101630)
Supplement: Supplementary file 1 [file pharmaceutics-13-01630-s001.zip › Supplementary Materials File S1 Analytical methodology for quantification of Colistin in Plasma .pdf]

pharmaceutics

MDPI

Supplementary Materials File S1: Estimation of the Difference in Colistin Plasma Levels in Critically Ill Patients with Favorable or Unfavorable Clinical Outcomes

Jose Sanabria, Vivian Garzón, Tatiana Pacheco, Maria-Paula Avila, Julio-Cesar Garcia, Diego Jaimes, Angela Torres, Rosa-Helena Bustos, Javier Escobar-Perez and Deisy Abril

1. Chemical and Materials

Colistin sulfate (purity: 99.8%) was used as an internal standard (IS), and obtained from Sigma Aldrich (St. Louis, MO, USA). The IS was stored between 2–8 °C. Other reagents, such as acetonitrile (ACN) of HPLC gradient grade, were purchased from Fisher Scientific (Leicestershire, UK), and ultra-pure water (HPLC grade, 18.2 MU.cm) was obtained by means of a Milli-Q water apparatus from Millipore (Milford, MA, USA). Reagents such as 1N Chloride Acid 1N, 1N sodium hydroxide (used for pH adjustment of the mobile phase), sodium phosphate monobasic monohydrate (NaH2PO4.H2O), and sodium phosphate dibasic (Na2HPO4) were used for the mobile phase, and were obtained from Sigma Aldrich (St. Louis, MO, USA).

2. Instrumentations and Chromatographic Conditions

The analysis was performed on a Hitachi LaChrom Elite HPLC system (Tokio, Japan) equipped with an auto sampler (Hitachi LaChrom Elite L-2200), column oven (Hitachi LaChrom Elite L-2300), HPLC pump (Hitachi LaChrom Elite L-2130), degasser, thermostatted sample reservoir, thermostatted column compartment, and UV detector (Hitachi LaChrom Elite L-7455). A Gemini® 5 µm, C18 column (150 x 4.6 mm from Torrance, USA) was used as the analytical column. The mobile phase was composed of solvent A: 0.2 M phosphate buffer at pH 6.5 and solvent B: ACN with a lineal gradient elution 20 to 25% B in A in 10 min, and a flow rate of 1.5 mL/min. The plasma samples were detected using a UV detector with the wavelength at 214 nm. The sample reservoir and column oven were maintained at 25°, and the injection volume was 10 µL. Instrumentation control and data acquisition were achieved with OpenLAB software. Several studies on colistine analysis were consulted for the validation procedures [1-5]

3. Sample preparation

The sample preparations for quantification of colistine concentrations were obtained according to a modified protocol reported in the literature [1-5]. For the validation of the methodology, we tested different concentrations of the antibiotic, changes in pH in the buffer, flow, wavelength, plasma dilution, etc., to find the best parameters for the quantification of the antibiotic.

The plasma samples were prepared with 40 µL of plasma of patients, 30 µL of 0.2 M phosphate buffer at pH 6.5, and 10 µL IS (colistine sulphate 2 mg/mL) and were mixed thoroughly. The aqueous supernatant (approximately 100 µL) was collected as the final sample, after centrifugation at 2500 g x 5 min. The plasma samples of the patients were analyzed in triplicate, and as a control the plasma sample was injected without enrichment with the IS. The final concentration of spiked colistine was 0.02 mg in 80 µL of sample (250 µg/mL).

4. Validation

The method validation was carried out with bioanalytical method validation guidance from ICH [6,7].

4.1. Preparation of the calibration curve, quality control, & internal standard (linearity)

Colistin sulfate stock solution for the standard curve and control, containing 2 mg/mL, was prepared in water. The working solutions were serially diluted from the stock solutions with 125 µL of control plasma and phosphate buffer. The final volume was 250 µL.

The calibration curve with total colistin concentrations in plasma (x) vs. the area of colistin B peak (y) was constructed for the plasma control using seven concentrations in the range of 10–1000 µg/mL. The calibration curve was validated over three different occasions. The LLOQ was defined as the lowest concentration on the calibration curve, with a precision of ≤20% coefficients of variation (CV) and an accuracy of 80–120%. The signal to noise ratio at the lower limit of quantification (LLOQ) was no less than 5. The calibration curve and quality control samples were prepared by spiking 10 µL of the working solution in 190 µL drug-free plasma. The peak area ratios versus concentrations were fitted into a linear regression curve: y=mx+b (least-square linear regression methods) using the OriginPro program (v.8.5). The figure S1, shows de calibration curve obtained.

4.2. Precision and accuracy

The precision of the peak area ratios and the accuracy were evaluated by analyzing analyte samples of known concentrations. The low, middle, and high concentration quality control samples (QCL, QCM and QCH) in plasma were 10, 100, 500 µg/mL. The intra- and inter-batch precision and accuracy were evaluated from three different batches by three levels of quality controls, and the LLOQ within the same day, for six replicates (n=6). These results are presented as CVs (acceptable if <5%).

4.3. Recovery and Matrix Effect

The matrix effect and extraction recovery were evaluated with the three levels of quality control concentrations for plasma. The matrix effect was evaluated with six different individuals. The recovery of the sample preparations was calculated by dividing the peak area for the pre-spiked sample by the peak area for the post-spiked sample, multiplied by 100 (%).

5. Results

5.1. Optimization of parameters of the HPLC method and IS

The parameters of the bioanalytical assay of colistin in the plasma samples were optimized to develop a precise and accurate HPLC method. The HPLC conditions described previously in plasma samples [2] were utilized as a backbone to devise our analytical method with IS. The effect of varying the pH of the mobile phase and composition was investigated at pH values 3.0, 6.5, and 7.4. The column efficiency decreased with an increase in pH. The buffer of sodium phosphate was the optimal buffer at a pH of 6.5. Chromatography HPLC was optimized by varying the composition of ACN (acetonitrile) and buffer phosphate (mobile phase) to eliminate interference between the colistin peak and the endogenous peaks of human plasma, for optimal separation of each peak. Endogenous interference was substantially attenuated by adjusting the mobile phase at pH 6.5. After evaluating a series of various mobile phase compositions, the final mobile phase for optimal conditions of separation of colistin was solvent A: 0.2 M phosphate buffer and solution B: ACN using a lineal gradient 20 to 25% of B in A in 10 min (Table S1).

Table S1. Gradient elution for the quantification of total colistin in human plasma samples.

Time (min)	Mobile phase composition	Flow rate (mL/min)
0	ACN: Buffer: Water, (20:80:0 v/v)	1.5
10	ACN: Buffer: Water, (25:75:0 v/v)	1.5
11	ACN: Buffer: Water, (0:50:50 v/v)	1.5
14	ACN: Buffer: Water, (0:50:50 v/v)	1.5

15	ACN: Buffer: Water, (0:0:100 v/v)	1.5
18	ACN: Buffer: Water, (0:0:100 v/v)	1.5
19	ACN: Buffer: Water, (20:80:0 v/v)	1.5
22	ACN: Buffer: Water, (20:80:0 v/v)	1.5

The UV detector wavelength was set at 214 nm to optimize the sensitivity for detection of the major peaks of colistin (colistin A and colistin B), which is consistent with the previously used UV wavelengths between 210 nm and 220 nm for colistin [2,5]. The retention times (RTs) of the major peak of colistin A and colistin B were shifted to avoid interference. The two peaks (colistin A and colistin B) were separated at different RTs of ~6 min and ~9 min. The peak of colistin B was used for the quantification of human plasma colistin (Figure S2). Our final optimized HPLC method adequately separated the major peak of colistin in human plasma samples.

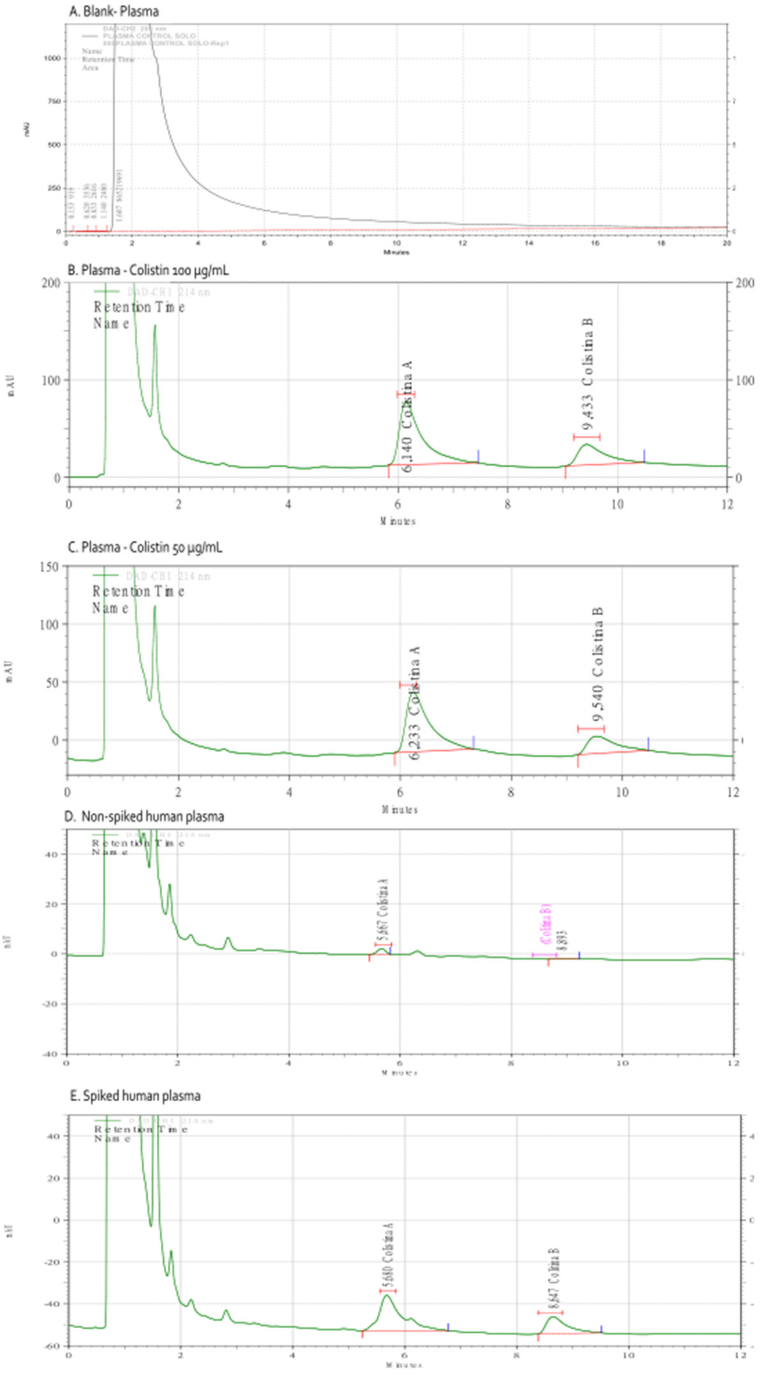

Figure S1. Chromatograms obtained via HPLC-ultraviolet detection of Colistin.

The linearity range of the method was between 1–1000 μg/mL of total colistin in the sample. In this method, patient samples enriched with 250 μg/mL of colistin were analyzed, and the final concentration of colistin in plasma was calculated from the total area minus the area of the 250 μg/mL standard sample. In this way, it was possible to determine colistin concentrations in the plasma of patients at a range of concentration of 0.1 - 10 μg/mL. The chromatograms of the blank with the internal standard, spiked samples containing 250 μg/mL colistin sulfate, and clinical samples are shown in Figure S1.

5.2. Validation of the optimized method: linearity, accuracy, precision, and recovery

Colistin (colistin A and colistin B) were eluted with a total run time of 10 min. The peaks of colistine were free of interfering peaks. The LLOQ was 1.2 μg/mL, and the calibration curve showed linearity (y=6967x-201428, r^2=0.99579). Figure S2 shows the calibration curve of colistin B.

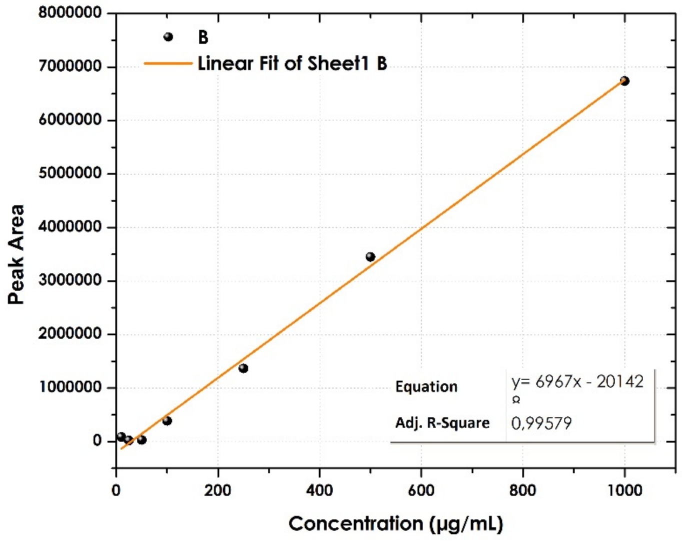

Figure S2. Calibration Curve of Colistin Standard.

The within- and between-run accuracy and precision range were in the range 95,3-108,5% and 5,2-9,5% respectively. These results are in accordance with the ICH guidelines [6,7] for bioanalytical methods. In terms of recovery, the mean standard deviation recovery was 102,4± 12,3%.

Considering the results reported in the literature on plasma concentrations of colistin in human plasma in the range of 0.1 – 10 μg/mL, the method validated in spiked colistin in plasma in this study was adequate for the plasma quantification of colistin.

References

1. Bai, L.; Ma, Z.; Yang, G.; Yang, J.; Cheng, J. A Simple HPLC Method for the Separation of Colistimethate Sodium and Colistin Sulphate. *Journal of Chromatography & Separation Techniques* **2011**, *2*, 1-4.
2. da Cunha-Pino, A. Desenvolvimento de uma técnica de HPLC para a quantificação de Colistina em plasma humano e a sua monitorização sérica em doentes internados no CHUC. *https://estudogeral.uc.pt/bitstream/10316/36563/1/DM%20Raquel%20Pinho.pdf* **2016**.
3. Hanai, Y.; Matsuo, K.; Kosugi, T.; Kusano, A.; Ohashi, H.; Kimura, I.; Hirayama, S.; Nanjo, Y.; Ishii, Y.; Sato, T., et al. Rapid, simple, and clinically applicable high-performance liquid chromatography method for clinical determination of plasma colistin concentrations. *Journal of Pharmaceutical Health Care and Sciences* **2018**, *4*.
4. Køppenn, B.; Bencic, N.; Melander, C. Characterization of colistimethate sodium (cms). 2014.

5. Liu, X.; Yu, Z.; Wang, Y.; Wu, H.; Bian, X.; Li, X.; Fan, Y.; Guo, B.; Zhang, J. Therapeutic drug monitoring of polymyxin B by LC-MS/MS in plasma and urine. *Bioanalysis* **2020**, *12*, 845-855.
6. Agency, E.M. Guideline on bioanalytical method validation. *https://www.ema.europa.eu/en/documents/scientific-guideline/guideline-bioanalytical-method-validation_en.pdf* **2011**.
7. Food and Drug Administration. Bioanalytical Method Validation Guidance for Industry. *https://www.fda.gov/regulatory-information/search-fda-guidance-documents/bioanalytical-method-validation-guidance-industry* **2018**.
