# Supplementary material for: Estimation of the Difference in Colistin Plasma Levels in Critically Ill Patients with Favorable or Unfavorable Clinical Outcomes
Source: Pharmaceutics. 2021 Oct 6;13(10):1630. doi: 10.3390/pharmaceutics13101630 (PMC8540821; doi:10.3390/pharmaceutics13101630)
Supplement: Supplementary file 1 [file pharmaceutics-13-01630-s001.zip › Supplementary Materials File S2 Description of Clinical Scales.pdf]

pharmaceutics MDPI

Supplementary Materials File S2 : Estimation of the Difference in Colistin Plasma Levels in Critically Ill Patients with Favorable or Unfavorable Clinical Outcomes

Jose Sanabria, Vivian Garzón, Tatiana Pacheco, Maria-Paula Avila, Julio-Cesar Garcia, Diego Jaimes, Angela Torres, Rosa-Helena Bustos, Javier Escobar-Perez and Deisy Abril

1. SOFA Scale

The Sequential Organ Failure Assessment (SOFA) Score is a mortality prediction score that is based on the degree of dysfunction of six organ systems. It is believed to provide a better stratification of the mortality risk in ICU patients given that the data used to calculate the score is not restricted to admission values. The SOFA Score can be used to determine level of organ dysfunction and mortality risk in ICU patients.

SOFA score	1	2	3	4
Respiration<br>PaO_2/FiO_2, mmHg	<400	<300	<200<br>— with respiratory support —	<100<br>— with respiratory support —
Coagulation<br>Platelets $\times 10^3/mm^3$	<150	<100	<50	<20
Liver<br>Bilirubin, mg/dl<br>(μmol/l)	1.2–1.9<br>(20–32)	2.0–5.9<br>(33–101)	6.0–11.9<br>(102–204)	>12.0<br>(<204)
Cardiovascular<br>Hypotension	MAP < 70 mmHg	Dopamine ≤5<br>or dobutamine (any dose)[a]	Dopamine >5<br>or epinephrine ≤0.1<br>or norepinephrine ≤0.1	Dopamine >15<br>or epinephrine >0.1<br>or norepinephrine >0.1
Central nervous system<br>Glasgow Coma Score	13–14	10–12	6–9	<6
Renal<br>Creatinine, mg/dl<br>(μmol/l) or urine output	1.2–1.9<br>(110–170)	2.0–3.4<br>(171–299)	3.5–4.9<br>(300–440)<br>or <500 ml/day	>5.0<br>(>440)<br>or <200 ml/day

[a] Adrenergic agents administered for at least 1 h (doses given are in μg/kg·min)

Interpretation
- Score 0 - 1 : Mortality 1,2%
- Score 1.1 - 2 : Mortality 5.4%
- Score 2.1 - 3 : Mortality 20%
- Score 3.1 - 4 : Mortality 36.1%
- Score 4.1 - 5 : Mortality 73.1%
- Score > 5.1 : Mortality 84.4%

References

- Vincent JL, Moreno R, Takala J, Willatts S, De Mendonça A, Bruining H, Reinhart CK, Suter PM, Thijs LG. The SOFA (Sepsis-related Organ Failure Assessment) score to describe organ dysfunction/failure. On behalf of the Working Group on Sepsis-Related Problems of the European Society of Intensive Care Medicine. Intensive Care Med. 1996 Jul;22(7):707-10. doi: 10.1007/BF01709751. PMID: 8844239.
- Ferreira FL, Bota DP, Bross A, Mélot C, Vincent JL. Serial evaluation of the SOFA score to predict outcome in critically ill patients. JAMA. 2001 Oct 10;286(14):1754-8. doi: 10.1001/jama.286.14.1754. PMID: 11594901.

2. APACHE II Score

This score can be calculated on all patients newly admitted to the intensive care unit. While it is not mandatory and will not help with patient management, it is a useful tool for risk stratification and to compare the care received by patients with similar risk characteristics in different units. Mortality prediction scores such as APACHE II are often used to assess the baseline risk groups being compared in clinical trials. They can also be used to determine prognosis and help family members make informed decisions about the aggressiveness of care.

PHYSIOLOGIC VARIABLE	HIGH ABNORMAL RANGE							LOW ABNORMAL RANGE	
	+4	+3	+2	+1	0	+1	+2	+3	+4
TEMPERATURE — rectal (°C)	≥ 41°	39°-40.9°		38.5°-38.9°	36°-38.4°	34°-35.9°	32°-33.9°	30°-31.9°	≤ 29.9°
MEAN ARTERIAL PRESSURE — mm Hg	≥ 160	130-159	110-129		70-109		50-69		≤ 49
HEART RATE (ventricular response)	≥ 180	140-179	110-139		70-109		55-69	40-54	≤ 39
RESPIRATORY RATE — (non-ventilated or ventilated)	≥ 50	35-49		25-34	12-24	10-11	6-9		≤ 5
OXYGENATION: A-aDO_2 or PaO_2 (mm Hg) a. FIO_2 ≥ 0.5 record A-aDO_2	≥ 500	350-499	200-349		< 200				
b. FIO_2 < 0.5 record only PaO_2					PO_2 > 70	PO_2 61-70		PO_2 55-60	PO_2 < 55
ARTERIAL pH	≥ 7.7	7.6-7.69		7.5-7.59	7.33-7.49		7.25-7.32	7.15-7.24	< 7.15
SERUM SODIUM (mMol/L)	≥ 180	160-179	155-159	150-154	130-149		120-129	111-119	≤ 110
SERUM POTASSIUM (mMol/L)	≥ 7	6-6.9		5.5-5.9	3.5-5.4	3-3.4	2.5-2.9		< 2.5
SERUM CREATININE (mg/100 ml) (Double point score for **acute** renal failure)	≥ 3.5	2-3.4	1.5-1.9		0.6-1.4		< 0.6		
HEMATOCRIT (%)	≥ 60		50-59.9	46-49.9	30-45.9		20-29.9		< 20
WHITE BLOOD COUNT (total/mm3) (in 1,000s)	≥ 40		20-39.9	15-19.9	3-14.9		1-2.9		< 1
GLASGOW COMA SCORE (GCS): Score = 15 minus actual GCS									
[A] Total ACUTE PHYSIOLOGY SCORE (APS): Sum of the 12 individual variable points									
Serum HCO_3 (venous-mMol/L) [Not preferred, use if no ABGs]	≥ 52	41-51.9		32-40.9	22-31.9		18-21.9	15-17.9	< 15

[B] AGE POINTS:
Assign points to age as follows:

AGE(yrs)	Points
≤ 44	0
45-54	2
55-64	3
65-74	5
≥ 75	6

[C] CHRONIC HEALTH POINTS
If the patient has a history of severe organ system insufficiency or is immuno-compromised assign points as follows:
a. for nonoperative or emergency postoperative patients — 5 points
or
b. for elective postoperative patients — 2 points

DEFINITIONS
Organ Insufficiency or immuno-compromised state must have been evident **prior** to this hospital admission and conform to the following criteria:

LIVER: Biopsy proven cirrhosis and documented portal hypertension; episodes of past upper GI bleeding attributed to portal hypertension; or prior episodes of hepatic failure/encephalopathy/coma.

CARDIOVASCULAR: New York Heart Association Class IV.

RESPIRATORY: Chronic restrictive, obstructive, or vascular disease resulting in severe exercise restriction, i.e., unable to climb stairs or perform household duties; or documented chronic hypoxia, hypercapnia, secondary polycythemia, severe pulmonary hypertension (>40mmHg), or respirator dependency.

RENAL: Receiving chronic dialysis.

IMMUNO-COMPROMISED: The patient has received therapy that suppresses resistance to infection, e.g., immuno-suppression, chemotherapy, radiation, long term or recent high dose steroids, or has a disease that is sufficiently advanced to suppress resistance to infection, e.g., leukemia, lymphoma, AIDS.

APACHE II SCORE
Sum of [A] + [B] + [C] :
[A] APS points ____________
[B] Age points ____________
[C] Chronic Health points ________
Total APACHE II ____________

FIG. 1. The APACHE II severity of disease classification system.

Interpretation
- Score 0 - 4 : Mortality 4%.
- Score 5 - 9 : Mortality 8%
- Score 10 - 14 : Mortality 15%
- Score 15 -19 : Mortality 25%
- Score 20 - 24 : Mortality 40%
- Score 25 - 29 : Mortality 55%
- Score 30 - 34 : Mortality73%
- Score >34 : Mortality 85%

References

- Knaus WA, Draper EA, Wagner DP, Zimmerman JE. APACHE II: a severity of disease classification system. Crit Care Med. 1985 Oct;13(10):818-29. PMID: 3928249.
- Headley J, Theriault R, Smith TL. Independent validation of APACHE II severity of illness score for predicting mortality in patients with breast cancer admitted to the intensive care unit. Cancer. 1992 Jul 15;70(2):497-503. doi: 10.1002/1097-0142(19920715)70:2<497::aid-cncr2820700220>3.0.co;2-h. PMID: 1617599.

3. Charlson Comorbidity Index

Calculates 10-year survival based on the patient's current comorbidities.

Weight	Clinical condition
1	Myocardial infarct<br>Congestive cardiac insufficiency<br>Peripheral vascular disease<br>Dementia<br>Cerebrovascular disease<br>Chronic pulmonary disease<br>Conjunctive tissue disease<br>Slight diabetes, without complications<br>Ulcers<br>Chronic diseases of the liver or cirrhosis
2	Hemiplegia<br>Moderate or severe kidney disease<br>Diabetes with complications<br>Tumors<br>Leukemia<br>Lymphoma
3	Moderate or severe liver disease
6	Malignant tumor, metastasis<br>Aids

Interpretation

- Score 0 : Survival to 10 years: 98%
- Score 1 : Survival to 10 years: 96%
- Score 2 : Survival to 10 years: 90%
- Score 3 : Survival to 10 years: 70%
- Score 4 : Survival to 10 years: 53%
- Score 5 : Survival to 10 years: 21%
- Score 6 : Survival to 10 years: 2%
- Score ≥ 7 : Survival to 10 years: 0%

References

- Charlson ME, Pompei P, Ales KL, MacKenzie CR. A new method of classifying prognostic comorbidity in longitudinal studies: development and validation. J Chronic Dis. 1987;40(5):373-83. doi: 10.1016/0021-9681(87)90171-8. PMID: 3558716.

- Quan H, Li B, Couris CM, Fushimi K, Graham P, Hider P, Januel JM, Sundararajan V. Updating and validating the Charlson comorbidity index and score for risk adjustment in hospital discharge abstracts using data from 6 countries. Am J Epidemiol. 2011 Mar 15;173(6):676-82. doi: 10.1093/aje/kwq433. Epub 2011 Feb 17. PMID: 21330339.
