# Supplementary material for: Estimation of the Difference in Colistin Plasma Levels in Critically Ill Patients with Favorable or Unfavorable Clinical Outcomes
Source: Pharmaceutics. 2021 Oct 6;13(10):1630. doi: 10.3390/pharmaceutics13101630 (PMC8540821; doi:10.3390/pharmaceutics13101630)
Supplement: Supplementary file 1 [file pharmaceutics-13-01630-s001.zip › Supplementary Materials File S3 Statistical analysis.pdf]

pharmaceutics

Supplementary Materials File S3: Estimation of the Difference in Colistin Plasma Levels in Critically Ill Patients with Favorable or Unfavorable Clinical Outcomes

Jose Sanabria, Vivian Garzón, Tatiana Pacheco, Maria-Paula Avila, Julio-Cesar Garcia, Diego Jaimes, Angela Torres, Rosa-Helena Bustos, Javier Escobar-Perez and Deisy Abril

SHAPIRO WILKS – NORMALITY TEST

```
. ***Anàlisis de Normalidad por subgrupos de acuerdo a descenlace

. by Desenlace_Favorable, sort : swilk Dias_hasta_inicio Edad Peso Talla IMC Conc_Colistina TFg_Dia1n TFG_Dia7n Días_hosp
```

-> Desenlace_Favorable = 0

Shapiro-Wilk W test for normal data

Variable	Obs	W	V	z	Prob>z
Dias_hasta~o	35	0.54102	16.382	5.837	0.00000
Edad	35	0.94397	2.000	1.447	0.07396
Peso	35	0.97668	0.832	-0.383	0.64916
Talla	35	0.92524	2.668	2.049	0.02024
IMC	35	0.96647	1.197	0.375	0.35389
Conc_Colis~a	23	0.74076	6.781	3.892	0.00005
TFg_Dia1n	31	0.95933	1.325	0.583	0.28010
TFG_Dia7n	27	0.89668	3.037	2.282	0.01124
Días_hosp	35	0.76814	8.276	4.411	0.00001

-> Desenlace_Favorable = 1

Shapiro-Wilk W test for normal data

Variable	Obs	W	V	z	Prob>z
Dias_hasta~o	50	0.66112	15.937	5.905	0.00000
Edad	50	0.97525	1.164	0.324	0.37298
Peso	50	0.98536	0.689	-0.796	0.78687
Talla	50	0.97377	1.233	0.447	0.32731
IMC	50	0.98216	0.839	-0.374	0.64592
Conc_Colis~a	34	0.47906	18.190	6.045	0.00000
TFg_Dia1n	48	0.95806	1.910	1.377	0.08424
TFG_Dia7n	36	0.94877	1.868	1.307	0.09565
Días_hosp	50	0.73092	12.654	5.413	0.00000

MULTIVARIATE ANALYSIS

REGRESION CON VAR SIGNIFICATIVAS********

```
logistic Desenlace_Favorable Genero SOFA_DÍA1 APACHE SOFA_DÍA7 Charlson Muerte30_días

ogistic regression                              Number of obs     =         85
                                                LR chi2(6)        =      27.62
                                                Prob > chi2       =     0.0001
og likelihood = -43.776687                      Pseudo R2         =     0.2398
```

esenlace_Favorable	Odds Ratio	Std. Err.	z	P>|z|	[95% Conf.	Interval]
Genero	.4389162	.2563752	-1.41	0.159	.1396969	1.379039
SOFA_DÍA1	.9771989	.4216326	-0.05	0.957	.4194831	2.276415
APACHE	.6522822	.2732452	-1.02	0.308	.2869867	1.48255
SOFA_DÍA7	.2853705	.1428622	-2.50	0.012	.1069745	.7612683
Charlson	.8893275	.2519087	-0.41	0.679	.5104492	1.549426
Muerte30_días	.617107	.6642266	-0.45	0.654	.0748453	5.088106
_cons	5.335646	2.402941	3.72	0.000	2.207207	12.89825
